# Supramolecular assemblies derived from methyl-substituted cucurbit[5]uril and lanthanide nitrates

**DOI:** 10.1016/j.heliyon.2024.e32936

**Published:** 2024-06-13

**Authors:** Shang Wei Yuan, Xue Dai, Ji Hong Lu, Pei Hua Ma, Scott J. Dalgarno, Carl Redshaw, Zhu Tao, Xin Xiao

**Affiliations:** a Key Laboratory of Macrocyclic and Supramolecular Chemistry of Guizhou Province, Guizhou University, Guiyang, 550025, China; b Institute of Chemical Sciences, School of Engineering and Physical Sciences, Heriot-Watt University, Edinburgh, EH14 4AS, UK; c Chemistry, School of Natural Sciences, University of Hull, Hull, HU6 7RX, UK

**Keywords:** Methylcucurbit[5]uril, Lanthanide, Supramolecular assembly

## Abstract

Interaction of the lanthanide nitrates M(NO_3_)_3_ (M = Gd, Eu) with methylcucurbit[5]uril (Me_10_Q[5]) in the presence of transition metal chlorides (ZnCl_2_ and FeCl_3_) in acidic media resulted in the isolation of the complexes [Me_10_Q[5]Gd(H_2_O)_2_Cl Gd(H_2_O)_6_](ZnCl_4_)_2_∙Cl∙8.9H2O (**1**) and [Me_10_Q[5]Eu(H_2_O)_3_Cl(H_3_O)](FeCl_4_)_3_ (**2**). The molecular structures of **1** and **2** have been determined by single crystal X-ray crystallography, and reveal discrete complexes which are involved in dense stacking with adjacent Me_10_Q[5]s linked via H-bonding and/or metal anions resulting in a supramolecular assembly.

## Highlights

• Molecular structures of lanthanide methylcucurcubit [5]uril (Me_10_Q [5]) complexes.
• Supramolecular assemblies formed via linking Me_10_Q [5] with H-bonds and/or metal anions.
• Use of metal chlorides as structure inducers/crystallisation agents.

## Graphical abstract

**Figure:**
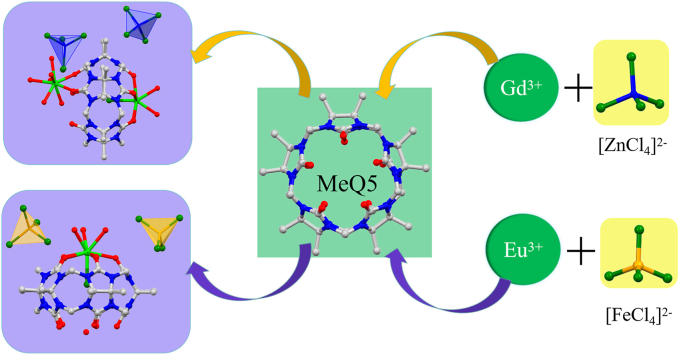

## 1 Introduction

In the field of supramolecular chemistry and crystal engineering, host-guest supramolecular self-assembly involving metal ions has received extensive attention, and has seen application in fields such as sensors, environmental pollution, catalysis, magnetic materials and micro-reaction vessels, etc. [1,2]. Cucurbit[*n*]urils (n = 5–8,10,13–15 abbreviated as Q[*n*]s), are a well-known family of macrocyclic compounds [3], and are receiving extensive attention in the field of supramolecular chemistry [4]. Indeed, recent work by Seth, Chopra *et al*. has studied the different noncovalent interactions utilized in forming various supramolecular networks, and has reported a quantitative analysis in the crystalline state [[5], [6], [7], [8], [9]]. For example, in substituted coumarin derivatives, in the molecular packing the various interactions such as C–H⋯O, π ^…^ π and carbonyl ^…^ π interactions participate in a cooperative fashion [6].

Q[*n*]s are an ideal organic scaffold for the complexation of metal ions in aqueous solution because the two sides have the same polar carbonyl group.

Decamethylcucurubit[5]uril (Me_10_Q[5]) was structurally characterized by Stoddart et al. and is the earliest reported fully substituted cyclic pentamer of the cucurbituril family [10]. The use of the dimethyl substituted glycosides instead of ordinary glycosides, allowed for the instruction of methyl groups at the melon ring. However, despite the lack of improvement in solubility, its recognition ability and selectivity toward metal ions were significantly improved [[11], [12], [13]]. Two partially methyl-substituted cucurbit[*n*]urils (n = 5, 6) were later structurally characterized by Tao and coworkers [14]. Compared to its homologues, the five membered ring systems and derivatives thereof have the smallest cavity and can generally only contain small molecules such as methane, ethylene, methanol, O_2_, N_2_, acetone, etc. [15,16] Studies have shown that unsubstituted Q[5], as well as a lanthanide capped derivative, can selectively encapsulate ions such as chloride or nitrate ions [17,18]. Moreover, the dipolar carbonyl oxygen atom at the portals of Q[5] and Me_10_Q[5] are capable of chelating to metals, and this further promotes anion encapsulation [19]. By contrast, the coordinated chemistry of alkylcucurbit[5] urils (RQ[5]) is somewhat limited. Reports include Lindoy, Tao, Wei et al. who reported the formation of 3D fused 10-membered “bracelet” frameworks when employing RQ[5] (R = 1,2,4-hexamethyl or pentacyclopentano) upon interaction with KX (X = Cl, I) [20]. The work was then expanded to include R = dimethyl, 1,3-dicyclohexano, 1,2,4-tricyclohexano and 1,2,3-cyclohexano, and a number of RQ[5]K networks were structurally characterized [21]. The pentacyclopentano derivative (PCyHQ[5]) was also reacted with alkali metal cations (Li, Na, K, Rb, Cs), with and without [MCl_4_]^2-^ anions present (M − Zn, Cd). In the case of M = Na, with no anions present, a 1D coordination polymer incorporating capsules of PCyHQ[5] was isolated. When [MCl_4_]^2-^ anions were employed, they acted as linkers in alkali-PCyHQ[5] supramolecular chains, and in the case of Rb, a 2D fused six-membered bracelet was formed for M = Zn [22]. Chen, Tao et al. extended such studies by reacting the R = 1,2,3-hexamethyl derivative with alkali cations (Na^+^, K^+^, Rb^+^, Cs^+^) and isolated capsule-like complexes in the presence of [CdCl_4_]^2-^ anions [23]. Capsules and bowls were also isolated from reactions involving Me_10_Q[5] and alkali metal salts (Na^+^, K^+^, Rb^+^ Cs^+^) [24].

More recently, Ma et al. reported the interaction of PCyHQ[5] with a number of transition metal perchlorates, namely Fe(ClO_4_)_3_, Co(ClO_4_)_2_ and Ni(ClO_4_)_2_, and it was found that the metal coordinated with the PCyHQ[5] portal leading to either 1D supramolecular chains or discrete molecules [25]. Cao et al. have combined decamethylcucurbit [5]uril with Keggin-type polyoxometallates and have isolated train-like supramolecular structures [26], and in subsequent work reported the effect of anions on the formation of various supramolecular assemblies [27].

In terms of lanthanide chemistry, Zhu et al. have reported that a methyl-substituted Q[5], derived from 3α-methyl glycoluril, interacts with the lanthanides La^3+^, Ce^3+^, Pr^3+^ and Nd^3+^ in the presence of [CdCl_4_]^2-^ to form solid crystalline samples adopting a capsule-like structure; the lanthanide cations Sm^3+^ to Lu^3+^ failed to afford solid products and remained in solution [28]. Other studies by the same group led to the structural characterization of capsules of derived from Q[5], Me_2_Q[5], Me_5_Q[5], α,β,γ-Me_6_Q[5] or Me_10_Q[5] that were capable of including either a chloride or nitrate anion whilst being capped by a lanthanide (Gd, Nd, Dy, Y) or the alkaline earth metal Sr [29,30]. Thuéry has also reported Q[5]-based capsules capped by a lanthanide (Ce, Sm, Gd) on one side and potassium on the other, plus in the case of ytterbium, a one-dimensional polymer [31]. We also note that Rawat et al. have studied the coordination chemistry of Q[5] (and Q[7]) toward Eu(III), and the reactions were found to be entropy driven (dehydration of the reagents upon complexation) [32]. A number of other studies have investigated the interaction of lanthanide cations with various Q[6]s, including for the unsubstituted Q[6], a coordination polymer involving [{Nd(NO_3_)(H_2_O)_4_}_2_(NO_3_@Q[6])]^3+^ chains [33], for methyl-Q[6] the use of [CdCl_4_]^2-^ as a structure directing agent [34], and for cyclohexanocucurbit [6]uril, in the presence of HCl and CdCl_2_, a family of 1D coordination polymers incorporation lanthanides (La, Ce, Pr, Nd, Gd, Tb, Dy) have been structurally characterized for which a lanthanide contraction effect was observed [35]. The formation of networked rings and channels by cucurbiturils in the presence of metal ions (and organic cations) has been reviewed [36,37].

Herein, we report our findings on the combination of lanthanide nitrates M(NO_3_)_3_ (M = Gd and Eu) with methylcucurbit [5]uril (Me_10_Q[5]) in the presence of either ZnCl_2_ (M = Gd) or FeCl_3_ (M = Eu) in acidic media, and compare with previous results obtained using the nitrate where M = Ce.

## 2 Experimental

### 2.1 General materials

The chemicals, such as, Gd(NO_3_)_3_·6H_2_O, Eu(NO_3_)_3_·6H_2_O and ZnCl_2_ were purchased from commercial suppliers and were used without further purification; distilled water was always used in the experiments. Me_10_Q[5] were prepared and purified according to the published procedures or procedures developed in our laboratory [38,39].

### 2.2 Spectroscopic methods

Infrared spectroscopy recorded in Bruker Vertex 70 FT-IR infrared spectrometer (Bruker Instruments Ltd, Germany). Each complex and spectrally pure potassium bromide were dried in a vacuum drying oven and mixed at a ratio of about 1 : 200. The mixture was thoroughly ground and mixed in an agate mortar, and then the tablets were pressed on a pressure machine for sample preparation using the matching mold of an infrared spectrometer. The absorption/transmission spectrum was recorded by an infrared spectrometer with a scanning range of 400–4000 cm^−1^. The IR spectra are provided in the SI (Figs. S1 and S2).

For the ^1^H NMR spectroscopic experiments, the compound Me_10_Q[5] was prepared in D_2_O as a solution of 1.0 × 10^−3^ mol L^−1^ and the spectrum was recorded on a Varian INOVA-400 M NMR spectrometer at 20 °C. The complexes **1** and **2** are paramagnetic and did not afford informative NMR spectra. The ^1^H NMR spectrum of Me_10_Q[5] is provided in the SI (Fig. S3).

### 2.3 X-ray crystallography

Single crystal X-ray diffraction data were collected on a Bruker APEX II diffractometer operating at 273(2) K using Mo-Kα radiation; selected crystallographic data are given in Table 1. Using Olex2 [40], the structures were solved with the SHELXT [41] structure solution program using Intrinsic Phasing and refined with the SHELXL [42] refinement package using Least Squares minimization.

**Table 1:** Crystallographic data for **1** and **2**.

Complex	1	2
CCDC	2308681	2308682
Formula	C_40_H_83.8_Cl_10_Gd_2_N_20_O_26.9_Zn_2_	C_40_H_59_Cl_13_EuFe_3_N_20_O_14_
Formula weight	2075.21	1824.43
Crystal system	Monoclinic	Monoclinic
Space group	*P*2_1_/*c*	*P*2_1_/*n*
Unit cell dimensions		
*a* (Å)	13.5501(7)	15.9891(8)
*b* (Å)	24.6907(14)	26.8956(14)
*c* (Å)	21.2925(5)	16.7891(9)
*α* (°)	90	90
*β* (°)	92.534(2)	117.336(2)
*γ* (°)	90	90
*V* (Å^3^)	7116.7(7)	6413.7(6)
*Z*	4	4
Temperature (K)	272(2)	273(2)
Wavelength (Å)	0.71073	0.71073
Calculated density (g.cm^−3^)	1.937	1.889
Absorption coefficient (mm^−1^)	2.972	2.247
Crystal size (mm^3^)	0.28 × 0.22 × 0.2	0.26 × 0.12 × 0.08
*θ*(max) (°)	55.11	55.272
Reflections measured	66344	76793
Unique reflections	15274	14607
*R*_int_	0.0542	0.0596
Reflections with *F*^2^ > 2σ(*F*^2^)	12931	10991
Number of parameters	973	890
*R*_1_ [*F*^2^ > 2σ(*F*^2^)]	0.0406	0.0471
w*R*_2_ (all data)	0.0998	0.1288
GOOF, *S*	1.049	1.026
Largest difference peak and hole (e Å^−3^)	1.52 and −1.48	2.15 and −1.10

### 2.4 Preparation of complex **1**

Me_10_Q[5] (48.5 mg, 0.05 mmol) was put in a small vial and 10 mL of a HCl solution (3 mol/L) of Gd(NO_3_)_3_·6H_2_O (45.1 mg, 0.1 mmol) and ZnCl_2_ (13.6 mg, 0.1 mmol) was added. The mixture was stirred and heated until all solids completely dissolved. Then the mixture was stirred for 5 min, filtered, and the solution placed in a ventilation area for slow evaporation over a period of about three weeks to afford colourless crystals of complex **1**. Yield: 52 %. Anal. Calcd for **1**, C_40_H_83.8_Cl_10_Gd_2_N_20_O_26.9_Zn_2_: C, 23.13; H, 4.08; N,13.49 %. Found: C, 23.01; H, 4.26; N, 13.37 %. IR (cm^−1^): 3587w, 3571w, 3447 m, 3381w, 1742s, 1632s, 1484s, 1310s, 1267 m, 1198w, 1160w, 1073s, 964w, 915 m, 876 m, 833 m, 764s, 721w, 671 m, 592 m, 461w.

### *2.5* *Preparation of complex***2**

Me_10_Q[5] (48.5 mg, 0.05 mmol) was put in a small vial and 10 mL of a HCl solution (3 mol/L) of Eu(NO_3_)_3_·6H_2_O (45.1 mg, 0.1 mmol) and FeCl_3_ (13.6 mg, 0.1 mmol) was added. The mixture was stirred and heated until all solids completely dissolved. Then the mixture was stirred for 5 min, filtered, and the solution placed in a ventilation area for slow evaporation over a period of about three weeks to afford colourless crystals of complex **2**. Yield: 49 %. Anal. Calcd for **2**, C_40_H_59_Cl_13_EuFe_3_N_20_O_14_: C, 26.33; H, 3.26; N,15.36 %. Found: C, 26.45; H, 3.38; N, 15.02 %. IR (cm^−1^): 3483w, 2987w, 2858w, 2767w, 1727bs, 1637s, 1495bs, 1412s, 1312s, 12D72s, 1200w, 1163w, 1075 m, 966w, 915 m, 876 m, 835w, 763s, 720w, 673 m, 594 m.

## 3 Results and discussion

**Fig. 1:**
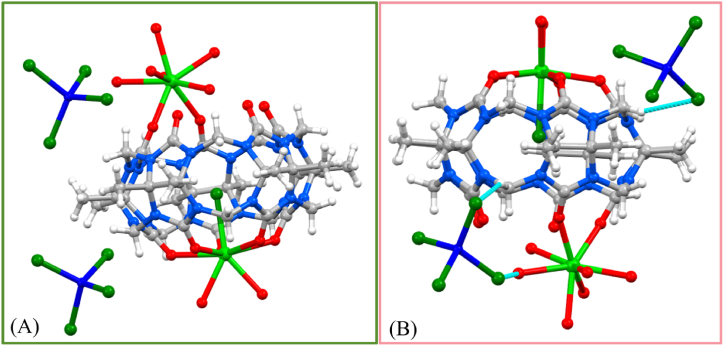
(A) Part of the asymmetric unit in **1** showing i) coordination of one (octa-coordinate) Gd^3+^ centre to all five carbonyl groups at one rim of a Me_10_Q[5] and the associated chloride counterion in the centre of the host cavity, ii) coordination of a second octa-coordinate Gd^3+^ centre to two of the carbonyl groups on the other Me_10_Q[5] rim, and iii) the presence of both chloride and ZnCl_4_ counterions providing charge balance. (B) The interaction forces and sites between two ZnCl_4^2−^_ ions and Me_10_Q[5]. Some H atoms and waters of crystallisation are omitted for clarity. Colour code: C – grey, N – blue, O – red, Cl – dark green, Zn – dark blue, Gd – light green. Dashed blue lines in B indicate H-bonding interactions between ZnCl_4^2−^_ ions and methylene Hs of the Me_10_Q[5] as per discussion.

Interaction of Me_10_Q[5] with an acidic (HCl) solution containing Gd(NO_3_)_3_·6H_2_O and ZnCl_2_, led following work-up, to the isolation of a white crystalline product in reasonable yield (*ca*. 52 %). Single crystals of **1** grown by slow evaporation under air were found to be in a monoclinic cell and structure elucidation was carried out in the space group *P*2_1_/*c*. The asymmetric unit (ASU, Fig. 1A and Fig. S4) contains the complex **1** and comprises one Me_10_Q[5] with a Gd^3+^ centre (Gd1) coordinating to all five carbonyl groups at one of the two rims (O1 – O5); five Gd–O bonds with distances in the range of 2.416(3) – 2.447(3) Å (Table 2). Gd1 also coordinates to an *endo*-cavity chloride ion (Cl1), and its coordination sphere is completed by two aquo ligands (O11 and O12, Table 2). Inspection of the other rim of the Me_10_Q[5] in Fig. 1 shows that Gd2 coordinates to two of the carbonyl groups (Gd-O6 and Gd-O7 with respective bond lengths of 2.313(3) – 2.370(3) Å), and its coordination sphere is completed by six aquo ligands (O13 – O18). Asymmetric capping has been observed in a number of other Q[*n*] systems [19,43].

**Table 2:** Bond lengths related to the coordination spheres of Gd1 and Gd2 in **1**.

Gd1	O1	2.416(3)	Gd2	O6	2.313(3)
Gd1	O2	2.424(3)	Gd2	O7	2.370(3)
Gd1	O3	2.416(3)	Gd2	O13	2.373(3)
Gd1	O4	2.446(3)	Gd2	O14	2.379(3)
Gd1	O5	2.447(3)	Gd2	O15	2.419(4)
Gd1	Cl1	2.6568(10)	Gd2	O16	2.410(3)
Gd1	O11	2.388(3)	Gd2	O17	2.445(3)
Gd1	O12	2.410(3)	Gd2	O18	2.409(3)

**Fig. 2:**
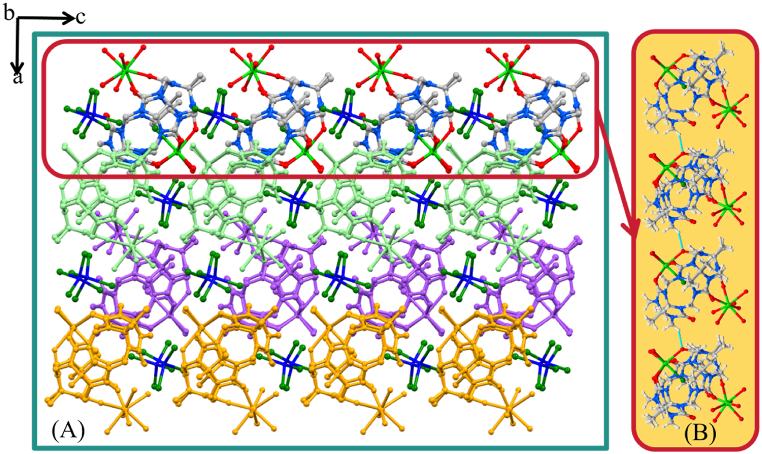
**(A)** Complex **1** stacked graph along the *b*-axis. **(B**) Extended structure in **1** showing the 1-D coordination polymer formed upon symmetry expansion, Colour code: C – grey, N – blue, O – red, Gd – light green. Dashed blue lines in B indicate H-bonding interactions between methylene hydrogens and carbonyl oxygens of symmetry equivalent Me_10_Q[5]s as per discussion.

Both Gd1 and Gd2 are octa-coordinate and possess square anti-prismatic geometries. Charge balance is provided through one Cl^−^ and two ZnCl_4^2−^_ ions, with one of the latter being disordered over two positions and modelled at partial occupancies. Within the structure, ZnCl_4^2−^_ ions are connected to the hydrogen on the methylene group of Me_10_Q[5] via ion dipole interactions, and also connected to the coordination water molecule of the Gd^3+^ cation (see Fig. 2B); the interaction distance is in the range 2.805–3.059 Å. In addition to this there are several waters of crystallisation in the lattice, some of which are associated with the aforementioned disorder in one of the ZnCl_4^2−^_ ions. We note that use of CdCl_2_·2.5H_2_O in acidic solutions with Q[6] leads to CdCl_4_^2−^ stabilized structures [[44], [45], [46]].

The layered stacking structure of the complex in the *b*-axis direction is shown in Fig. 2A. The stacking structure is very dense and has no obvious channels. In particular, the adjacent Me_10_Q[5]s are connected via hydrogen bonding interactions between the hydrogen on the bridged methylene and the carbonyl oxygen of Me_10_Q[5]; this interaction (C40–H40A … O3* occurs with a CH⋯O distance of 2.305 Å. The repeated basic chain structure is shown in Fig. 2B. The one-dimensional chain structure is continuously stacked in three dimensions to form the supramolecular assembly **1**. The non-integer water ratio in **1** relates to disorder in the structure that has been modelled as effectively as possible.

We note that a number of other supramolecular assemblies supported by non-covalent interactions have been reported recently. Examples include unusual 2D and 3D supramolecular networks derived on 2,6-pyridinedicarboxylic acid and cobalt(II) ions reported by Seth et al. The structures are stabilized by both H-bonding and a variety of π-π/π-π^+^ etc. type interactions [47]. The same group observed similar π-type interactions in pyridinium-carboxylate salts [48], whilst noncovalent interactions (C–H⋯O and O–H⋯O interactions) have also been reported in metal (Co, Cu) complexes derived from 1,3-diamino-2-hydroxypropanetetraacetate [49]. In some of these systems, Hirshfeld surfaces and 2D fingerprint plots [50] were employed to quantify the intermolecular interactions. Moreover, the noncovalent interactions were characterized by employing Bader's theory of ‘Atoms in Molecules’ [51].

**Fig. 3:**
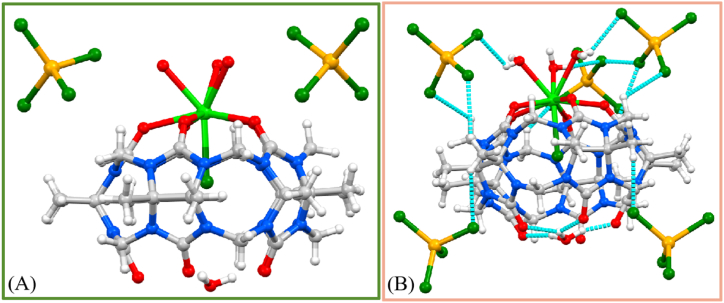
Part of the ASU in **2** showing **(A)** the presence of two FeCl_4^−^_ counterions providing charge balance, noting that these are shown in only one of two disordered positions for clarity, **(B)** a) coordination of the nona-coordinate Eu^3+^ centre to all five carbonyl groups at one rim of a Me_10_Q[5], the associated chloride counterion in the centre of the host cavity, and three aquo ligands; b) the presence of a H-bonded H_3_O^+^ cation at the other Me_10_Q[5] rim; Some H atoms (except those of the H_3_O^+^ cation) are omitted for clarity. The final FeCl_4_^−^ counterion could not be modelled appropriately and was handled using a mask. Colour code: C – grey, N – blue, O – red, Cl – dark green, Fe – dark yellow, Eu – light green. Hydrogen bonding and ion-dipole interactions are shown as dashed blue lines.

An analogous reaction using Eu(NO_3_)_3_·6H_2_O and FeCl_3_ in place of Gd(NO_3_)_3_·6H_2_O and ZnCl_2_ led, following work-up, to the isolation of a white crystalline product in reasonable yield (*ca*. 52 %). Single crystals of **2,** also grown by slow evaporation under, air were also found to be in a monoclinic cell and structure elucidation was carried out in the space group *P*2_1_/*n*. The ASU (Fig. 3A and Fig. S5) contains the complex **2** and comprises one Me_10_Q[5] with a Eu^3+^ centre (Eu1) coordinating to all five carbonyl groups at one of the two rims (O1 – O5, Table 3); five Eu–O bonds with distances in the range of 2.447(3) – 2.523(3) Å. Eu1 also coordinates to an *endo*-cavity chloride ion (Cl1), and its coordination sphere is completed by three aquo ligands below the coordinated Me_10_Q[5] rim (O11 – O13); bond lengths are listed in Table 3.

**Table 3:** Bond lengths related to the coordination sphere of Eu1 in **2**.

Eu1	O1	2.476(3)	Eu1	O11	2.461(3)
Eu1	O2	2.447(3)	Eu1	O12	2.480(4)
Eu1	O3	2.458(3)	Eu1	O13	2.473(3)
Eu1	O4	2.470(3)	Eu1	Cl1	2.7358(11)
Eu1	O5	2.523(3)			

Inspection of the other rim shows that an H_3_O^+^ cation (electron density in the difference map also suggested this, in addition to providing sensible charge balance) occupies the centroid of the penta-oxo rim, forming four hydrogen bonds with O(14) ^…^ O(6, 7, 8, 10) distances in the range of 2.526(3) – 2.667(3) Å (Fig. 3B and Fig. S6). This cation provides the charge counterbalance for **2**. The Eu1 centre is nona-coordinate and has tri-capped trigonal prismatic geometry. Charge balance is provided through three FeCl_4^−^_ ions, all of which are disordered over two or more positions. Two of these anions were successfully modelled at partial occupancy, but the third was severe and could not be handled effectively. Given this, a mask was applied to the data, and this markedly improved the agreement indices. As shown in Fig. 3B, within complex **2** the FeCl_4^−^_ anions interact via C–H⋯Cl ion-dipole interactions, and the interaction distances are in the range 2.459–2.946 Å. In addition to this, there are a number of waters of crystallisation in the lattice, some of which are associated with the aforementioned disorder in one of the FeCl_4^−^_ ions.

**Fig. 4:**
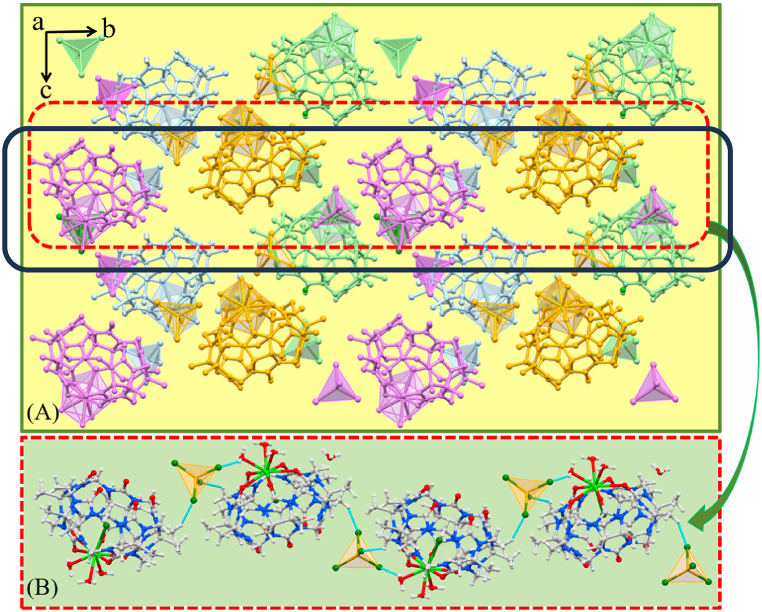
**(A)** Complex **2** stacked graph along the *a*-axis. **(B**) Extended structure in **2** showing the 1-D coordination polymer formed upon symmetry expansion, Colour code: C – grey, N – blue, O – red, Eu – light green, Fe – yellow. Dashed blue lines in B indicate interactions between symmetry equivalent Me_10_Q[5] methylene hydrogens and FeCl_4^−^_ anions.

As shown in Fig. 4A, the different orientations of the complexes are repeatedly stacked, and the stacking diagram in the *a* direction is constructed. It is observed that the structure is tightly packed and there are no obvious gaps. The FeCl_4^−^_ anions plays a very important role in the supramolecular assembly, and are connected to the outer wall of the Me_10_Q[5] (Fig. 4B); water molecules are coordinated to Eu^3+^ by ion dipole interactions.

**Fig. 5:**
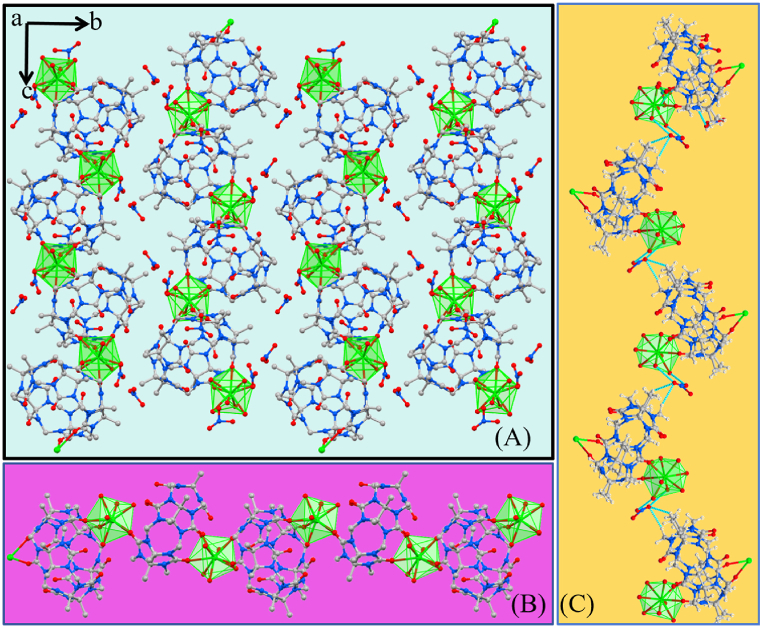
**(A)** Known complex **3** stacked graph along the *a*-axis. **(B, C**) Extended structure in **3** showing the 1-D coordination polymer formed upon symmetry expansion, Colour code: C – grey, N – blue, O – red, Eu – light green. Dashed blue lines in C indicate H-bonding interactions between symmetry equivalent aquo ligands and nitrate anions.

In contrast to the results above, previous results have shown that treatment of Me_10_Q[5] with an acidic (HNO_3_) solution of Ce(NO_3_)_3_·6H_2_O led, following work-up, to 1-D coordination polymer chains **3** (see Fig. 5) [52]. Hydrogen bonding also exists between the nitrate anions with the oxygen atoms of the coordinated (at Ce^3+^) water molecules. These multiple hydrogen bonds allow the 1-D coordination polymer chains to form a two-dimensional supramolecular structure. As shown in Fig. 5C, the extensive hydrogen bonding between nitrate ions and Me_10_Q[5] and Ce-coordinated water molecules leads to the construction of an S-type one-dimensional supramolecular chain. The assembly is composed of these interlaced long chains.

## 4 Conclusion

In conclusion, the interaction of the lanthanide nitrates M(NO_3_)_3_ (M = Gd, Eu) with methylcucurbit[5]uril in acidic media, in the presence of transition metal chlorides (ZnCl_2_ and FeCl_3_), led to the formation of two distinct complexes: [Me_10_Q[5]Gd(H_2_O)_2_Cl Gd(H_2_O)_6_](ZnCl_4_)_2_·Cl∙8.9H_2_O (**1**) and [Me_10_Q[5]Eu(H_2_O)_3_Cl(H_3_O)](FeCl_4_)_3_ (**2**). These complexes were isolated and their molecular structures elucidated by single crystal X-ray crystallography, revealing their discrete nature and involvement in supramolecular assemblies through dense stacking and intermolecular interactions. This is in contrast to the previously reported [Me_10_Q[5]Ce(H_2_O)_5_](NO_3_)_3_∙16H_2_O, which forms a coordination polymer rather than discrete complexes. The study highlights the influence of the presence of transition metal chlorides on the structural outcomes of the reactions, providing valuable insights into the design and synthesis of new supramolecular architectures.

## Data availability statement

The crystallographic data herein has been deposited in the Cambridge Structural Database, see numbers 2308681 and 2308682.

## CRediT authorship contribution statement

**Shang Wei Yuan:** Investigation. **Xue Dai:** Investigation. **Ji Hong Lu:** Methodology, Formal analysis. **Pei Hua Ma:** Methodology. **Scott J. Dalgarno:** Formal analysis. **Carl Redshaw:** Writing – review & editing, Writing – original draft, Funding acquisition. **Zhu Tao:** Methodology. **Xin Xiao:** Writing – review & editing, Writing – original draft, Supervision, Conceptualization.

## Declaration of competing interest

The authors declare that they have no known competing financial interests or personal relationships that could have appeared to influence the work reported in this paper.
