# Supplementary material for: Supramolecular assemblies derived from methyl-substituted cucurbit[5]uril and lanthanide nitrates
Source: Heliyon. 2024 Jun 13;10(13):e32936. doi: 10.1016/j.heliyon.2024.e32936 (PMC11261008; doi:10.1016/j.heliyon.2024.e32936)
Supplement: Multimedia component 1 [file mmc1.docx]

**Supporting Information: Supramolecular assemblies derived from methyl-substituted cucurbit[5]uril and lanthanide nitrates**

Shang Wei Yuan,^a^ Xue Dai,^a^ Ji Hong Lu,^a^ Pei Hua Ma,^a^ Scott J. Dalgarno,^b^ Carl Redshaw^c,*^ Zhu Tao,^a^ Xin Xiao ^a,*^

*^a^ Key Laboratory of Macrocyclic and Supramolecular Chemistry of Guizhou Province, Guizhou University, Guiyang 550025, China*

*^b^* *Institute of Chemical Sciences, School of Engineering and Physical Sciences, Heriot-Watt University, Edinburgh EH14 4AS, U.K.*

*^c^ Chemistry, School of Natural Sciences, University of Hull, Hull HU6 7RX, U.K.*

**Contents**

**Figure S1.** IR spectrum of **1**.

**Figure S2.** IR spectrum of **2**.

**Figure S3.** ^1^H NMR spectrum of Me_10_Q[5].

**Figure S4.** Thermal ellipsoid (50%) plot of the crystal structure of **1** with H atoms omitted for clarity. Colour code: C – grey, N – blue, O – red, Gd – light green, Zn – dark blue, Cl – dark green. H atoms omitted for clarity.

**Figure S5.** Thermal ellipsoid (50%) plot of the crystal structure of **2** with H atoms omitted for clarity. Colour code: C – grey, N – blue, O – red, Eu – light green, Fe – dark blue, Cl – dark green. H atoms omitted for clarity.

**Figure S6.** Part of the crystal structure of **2** showing H-bonding interactions between the H_3_O^+^ cation and carbonyl oxygens of the Me10Q[5]. Selected labels added to support discussion in the manuscript. Colour code: C – grey, N – blue, O – red, H – white, Eu – light green, Cl – dark green. Other H atoms and anions are omitted for clarity.


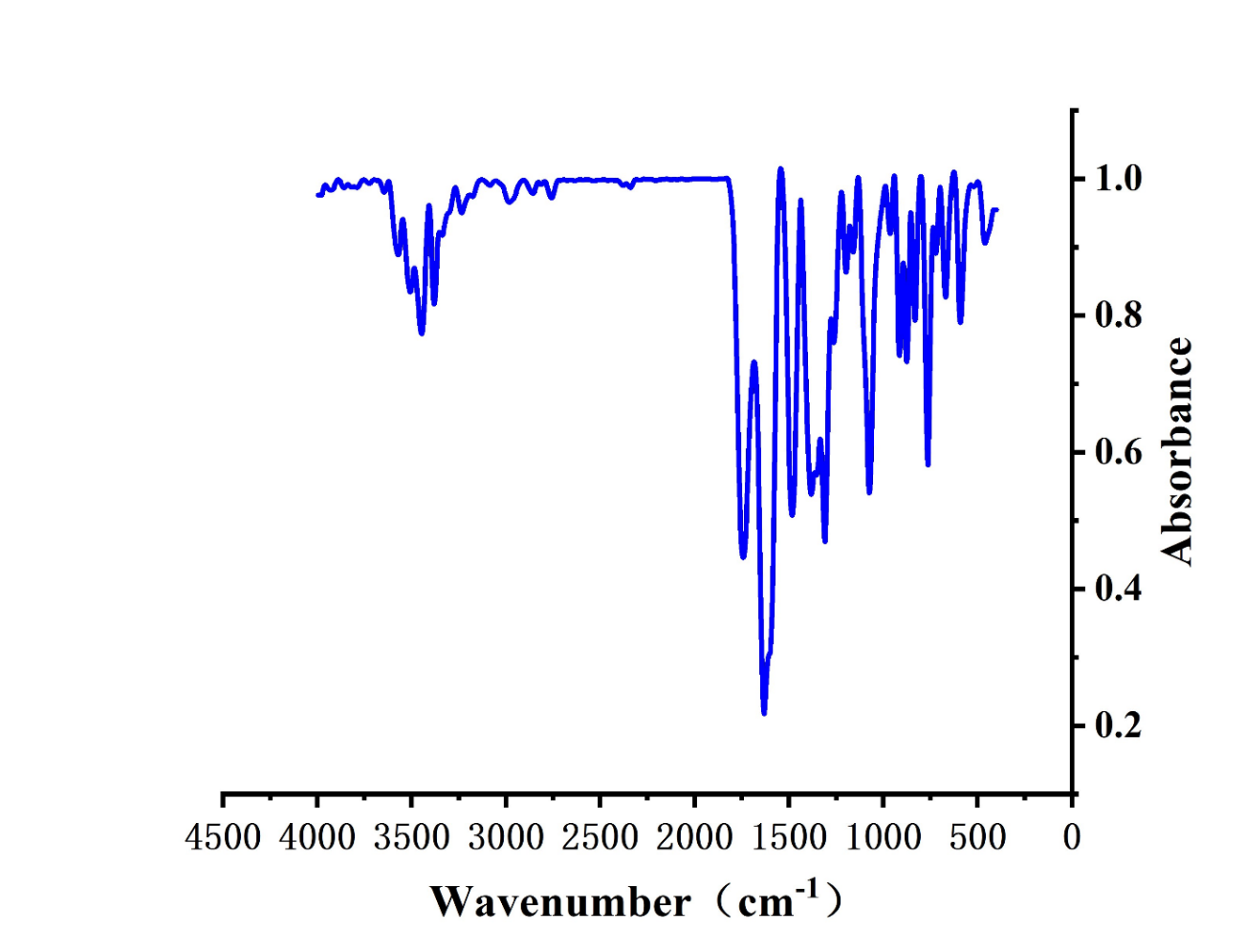
**Figure S1.** IR spectrum of **1**.


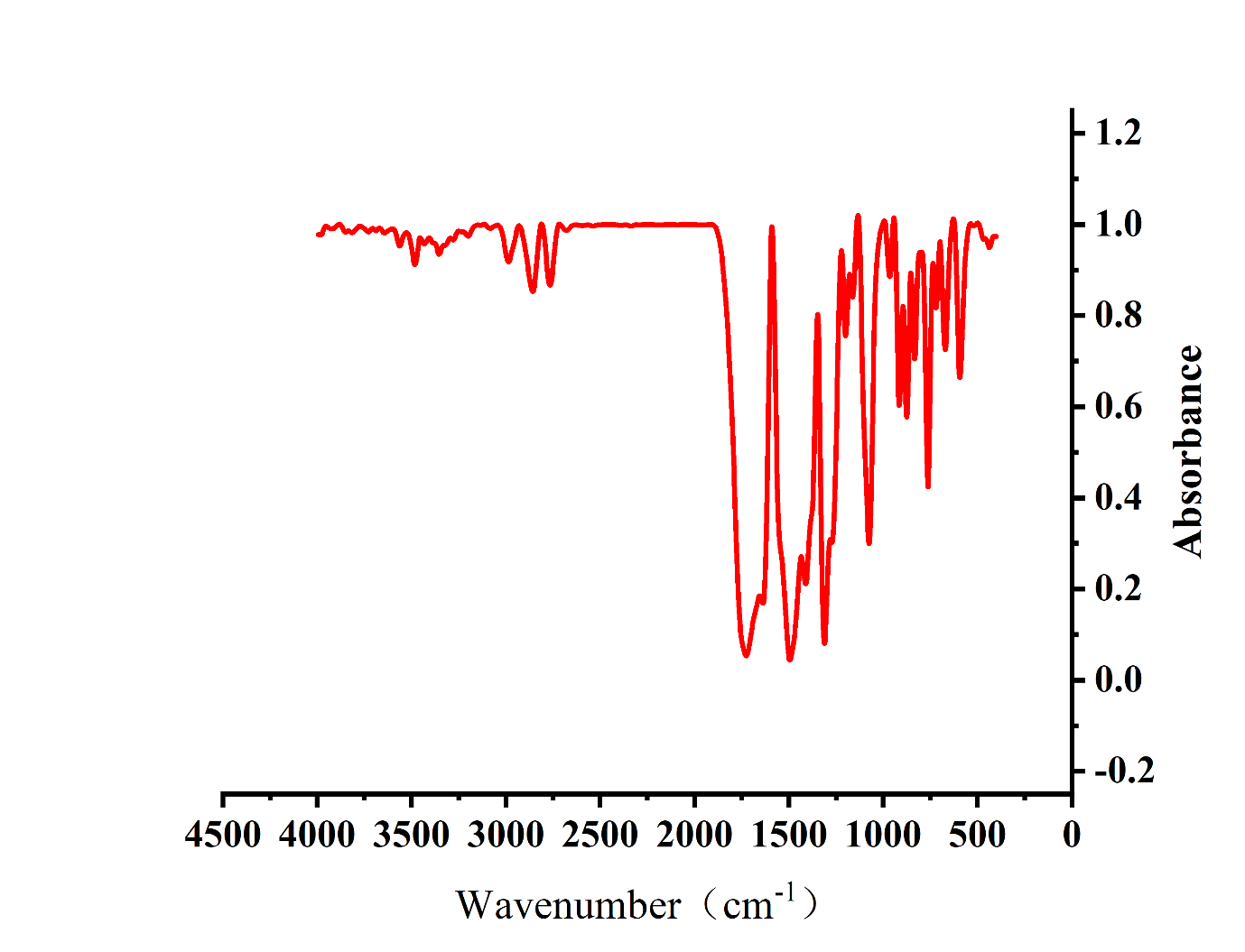


**Figure S2.** IR spectrum of **2**.


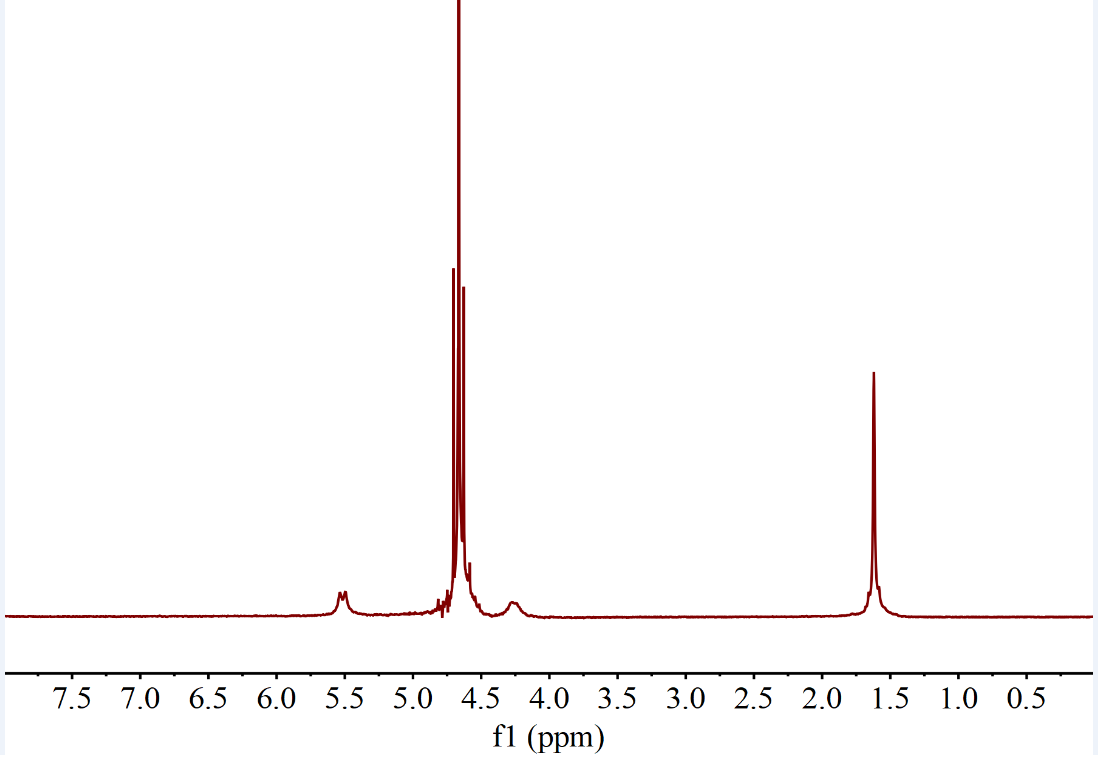


**Figure S3.** ^1^H NMR spectrum of Me_10_Q[5].


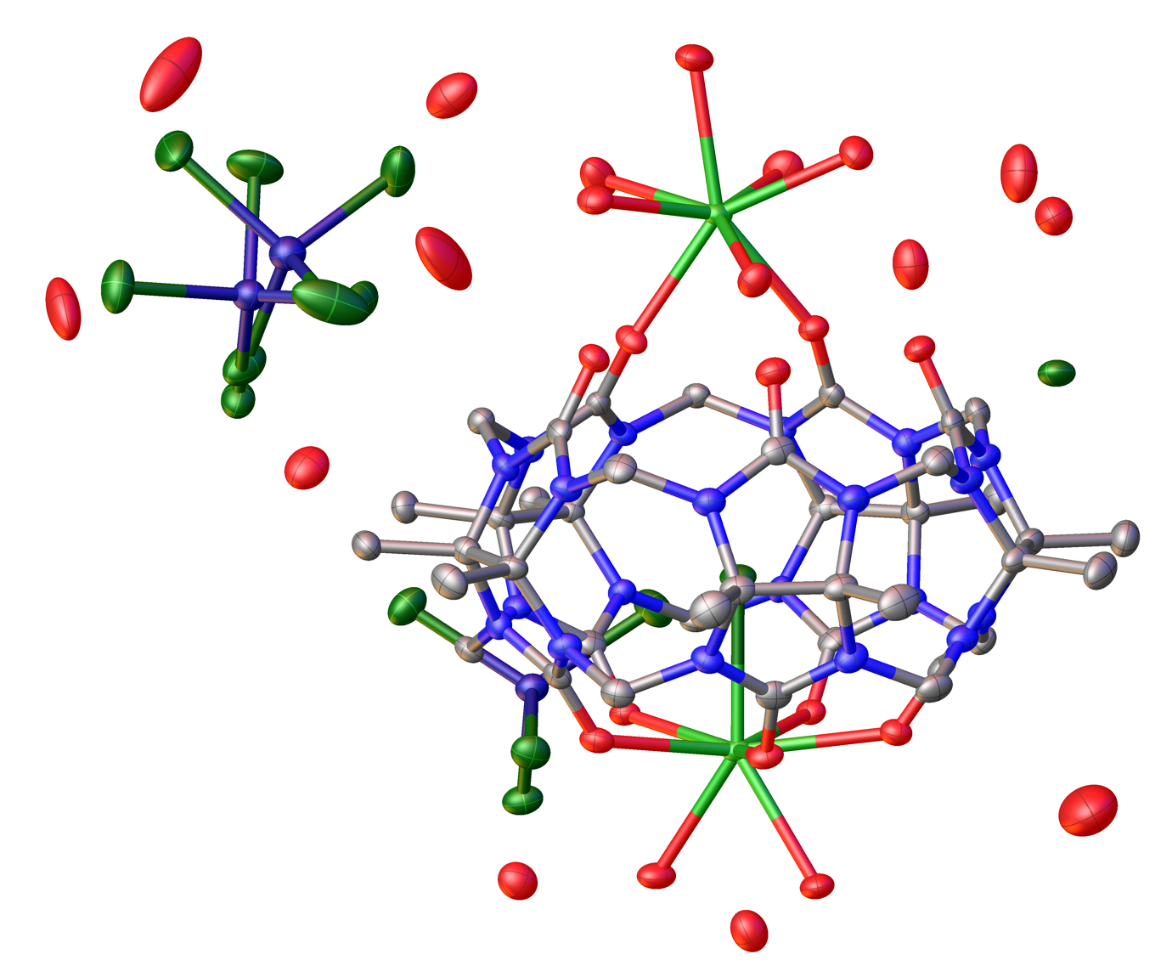


**Figure S4.** Thermal ellipsoid (50%) plot of the crystal structure of **1** with H atoms omitted for clarity. Colour code: C – grey, N – blue, O – red, Gd – light green, Zn – dark blue, Cl – dark green. H atoms omitted for clarity.


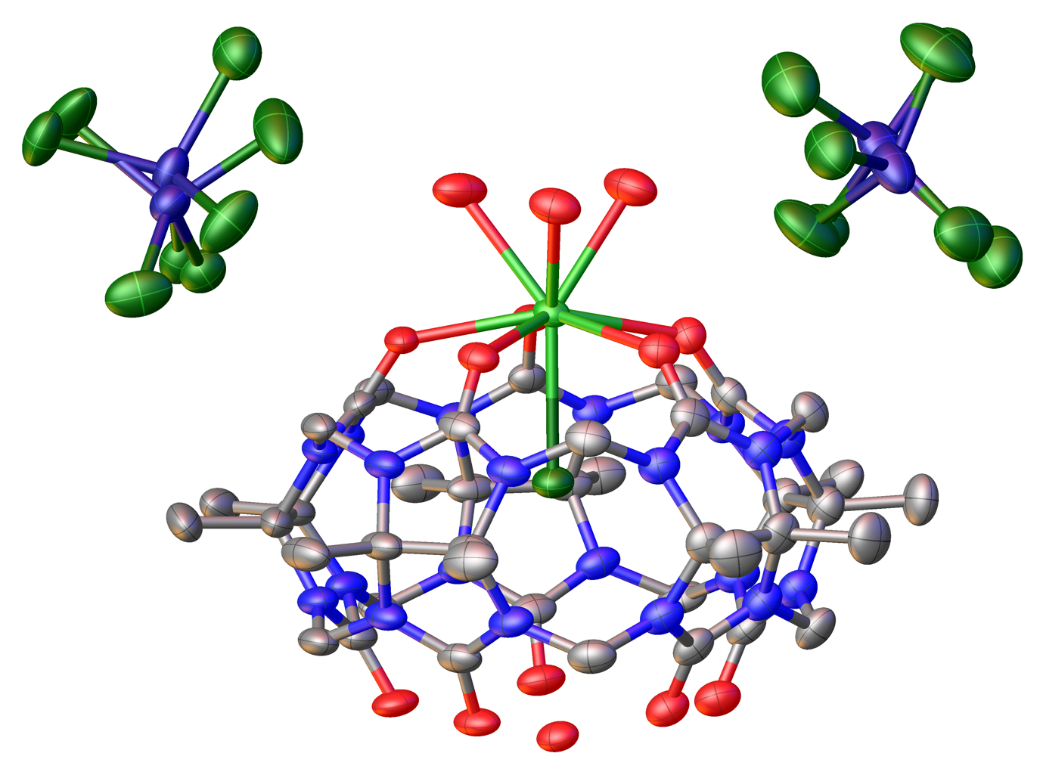


**Figure S5.** Thermal ellipsoid (50%) plot of the crystal structure of **2** with H atoms omitted for clarity. Colour code: C – grey, N – blue, O – red, Eu – light green, Fe – dark blue, Cl – dark green. H atoms omitted for clarity.


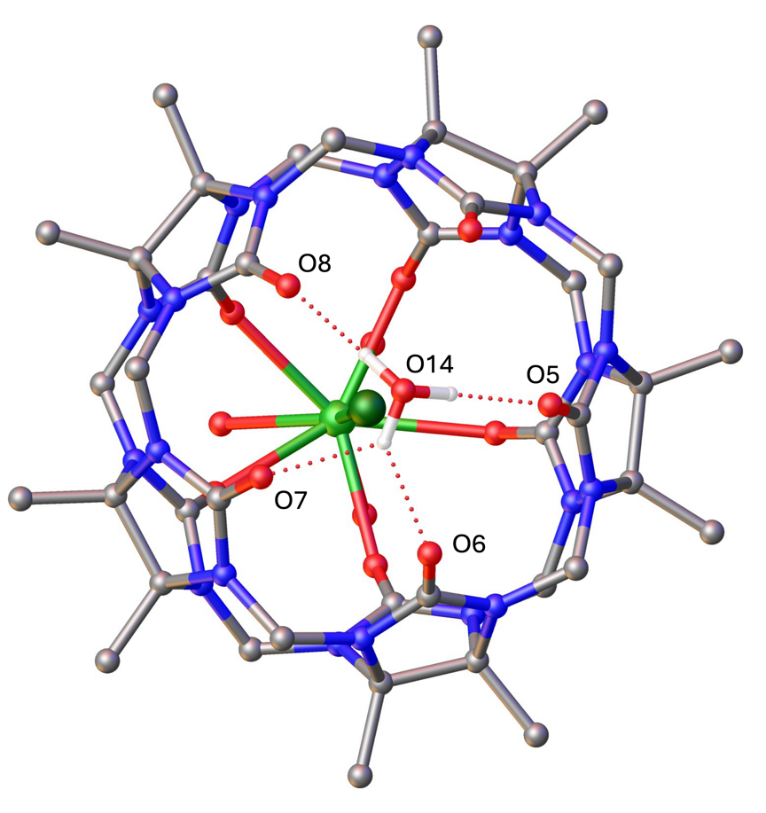


**Figure S6.** Part of the crystal structure of **2** showing H-bonding interactions between the H_3_O^+^ cation and carbonyl oxygens of the Me10Q[5]. Selected labels added to support discussion in the manuscript. Colour code: C – grey, N – blue, O – red, H – white, Eu – light green, Cl – dark green. Other H atoms and anions are omitted for clarity.
